# Tumor alkalization therapy: misconception or good therapeutics perspective? – the case of malignant ascites

**DOI:** 10.3389/fonc.2024.1342802

**Published:** 2024-02-08

**Authors:** Alexey Bogdanov, Nikolay Verlov, Andrey Bogdanov, Vladimir Burdakov, Valeriy Semiletov, Vitaliy Egorenkov, Nikita Volkov, Vladimir Moiseyenko

**Affiliations:** Napalkov Saint Petersburg Clinical Research and Practical Center of Specialized Types of Medical Care (Oncological), Saint Petersburg, Russia

**Keywords:** tumor acidity, alkalization therapy, malignant ascites, sodium bicarbonate, intraperitoneal perfusion

## Abstract

Tumor acidity has been identified as a key factor in promoting cancer progression, metastasis, and resistance. Tumor alkalization therapy has emerged as a potential strategy for cancer treatment. This article provides preclinical and clinical evidence for tumor alkalization therapy as a promising cancer treatment strategy. The potential of tumor alkalization therapy using sodium bicarbonate in the treatment of malignant ascites was studied. The concept of intraperitoneal perfusion with an alkalizing solution to increase the extracellular pH and its antitumor effect were explored. The significant extension in the overall survival of the Ehrlich ascites carcinoma mice treated with sodium bicarbonate solution compared to those treated with a sodium chloride solution was observed. In the sodium bicarbonate group, mice had a median survival of 30 days after tumor cell injection, which was significantly (p<0.05) different from the median survival of 18 days in the sodium chloride group and 14 days in the intact group. We also performed a case study of a patient with ovarian cancer malignant ascites resistant to previous lines of chemotherapy who underwent intraperitoneal perfusions with a sodium bicarbonate solution, resulting in a significant drop of CA-125 levels from 5600 U/mL to 2200 U/mL in and disappearance of ascites, indicating the potential effectiveness of the treatment. The preclinical and clinical results obtained using sodium bicarbonate perfusion in the treatment of malignant ascites represent a small yet significant contribution to the evolving field of tumor alkalization as a cancer therapy. They unequivocally affirm the good prospects of this concept.

## 1 Introduction

Tumor acidity is a hallmark of cancer that is associated with metabolic reprogramming and the use of glycolysis (Warburg effect), resulting in a high intracellular lactic acid concentration (1). This shift in the acid-base balance, including reversed pH-gradient of cancer cells (intracellular pH > extracellular pH), promotes various cancer characteristics, including proliferation, apoptosis avoidance, invasiveness, metastatic potential, aggressiveness, immune evasion, and treatment resistance (1–3). The acidic microenvironment around the tumor creates a positive feedback loop for carcinogenesis and encourages the selection of certain cell phenotypes that can survive in this environment. Acidosis has been recognized as a key chemical signature of the tumor microenvironment, and responses to acidosis give cancer cells a competitive advantage over the host (1–4). Understanding the basis of this advantage and finding ways to exploit tumor acidity therapeutically is an active area of research (1–5). Tumor alkalization therapy, which aims to neutralize/alkalize the acidic tumor microenvironment, has shown potential benefits in cancer treatment. Studies have reported that alkalization therapy, including an alkaline diet and/or oral alkalizing agents, can suppress cancer progression and enhance the effects of anti-cancer drugs (6–8). For example, in hepatocellular carcinoma (HCC) patients, the addition of alkalization therapy to standard treatments was associated with more favorable outcomes, with increased urine pH (≥ 7) after alkalization therapy being a potential marker for improved survival (7). Similarly, in patients with metastatic or recurrent pancreatic cancer, alkalization therapy, consisting of an alkaline diet and supplementary oral sodium bicarbonate, was found to enhance the effects of chemotherapy and improve overall survival (9, 10).

Buffer systems, such as sodium bicarbonate, can be also administered parenterally to directly neutralize tumor acidity. For example, not only oral but also intraperitoneal treatment with sodium bicarbonate reduced the pH gradient in mouse models of breast cancer, enhancing the response to weak base chemotherapy drugs (11). It was shown that sodium bicarbonate inhibits the growth of 4T1 breast cancer tumors in animal studies when its injected subcutaneously surrounding the tumors every other day (12). In our study, we examined the effects of a single perfusion using a 4% sodium bicarbonate solution on rat limbs with a Pliss lymphosarcoma graft. We found that the median survival period for the sodium bicarbonate group was 17 days, which was longer than both the nonperfused group and the isotonic saline group, which had median survival periods of 13 days (13). The clinical study ChiCTR-IOR-14005319 evaluated and compared the efficacies of conventional transarterial chemoembolization (TACE) and targeting intratumoral lactic acidosis TACE (TILA-TACE) with the local administration of a 5% sodium bicarbonate solution in patients diagnosed with large-focal hepatocellular carcinoma. The objective response rate (ORR) was 100% when sodium bicarbonate was employed, while the rate was 44.4% with conventional TACE in a nonrandomized cohort, and 63.6% in a randomized study (14). A recent case study reported that a 46-year-old man with Doege-Potter syndrome, secondary to liver and pancreatic metastatic solitary fibrous tumors, achieved clinical cure of hypoglycemia and well-controlled liver metastatic tumor after receiving six rounds of TILA-TACE treatment (15). These findings suggest that sodium bicarbonate solution may have potential therapeutic benefits for cancer treatment, although further studies are needed to confirm these results.

But why scientific and medical societies have expressed skepticism about the use of alkalization therapy for cancer treatment (16). The current state of research in this field indicates that the antitumor, antimetastatic, and immunotherapy-enhancing effects of buffer therapy have been mostly studied as a phenomenon (1). However, there is a lack of interest from pharmaceutical companies, possibly due to the lower cost of drugs like sodium bicarbonate (17) compared to chemotherapy, targeted, or immunotherapy drugs. Additionally, skepticism within the scientific and medical community is influenced by the “folk” use of baking soda and the presence of unreliable information on the internet. Furthermore, the lack of personal knowledge about tumor acidification mechanisms and alkalization possibilities, as well as the limited availability of reliable data, contribute to this skepticism. More research is needed to address these concerns and further explore the potential of buffer therapy in cancer treatment (1, 18, 19). Indeed, while the improved chemotherapeutic effect of weak base drugs can be explained by “ion trapping,” (20, 21) the broader effects of buffered therapy are complex and require further investigation. Buffering of the tumor microenvironment can impact enzymes involved in tumor invasion (22) and reduce PD-L1 expression (23), potentially enhancing immunotherapy. Additionally, neutralization of lactic acid with sodium bicarbonate can reactivate metabolically altered T cells (24). Anyway, it is important to note that alkalization therapy does not contradict but can be used in combination with standard treatment methods to potentially increase effectiveness (8).

In this paper, based on our data, we aim to discuss the perspectives of sodium bicarbonate treatment of malignant ascites. Malignant ascites is a common complication in patients with peritoneal carcinomatosis (25). It is the accumulation of fluid in the peritoneal cavity due to cancer and often signifies the terminal phase of the disease. The management of malignant ascites remains a clinical challenge, and symptom palliation is the current standard of care (25, 26).

## 2 Malignant ascites therapy with sodium bicarbonate: preclinical studies

We investigated the antitumor effects of a single intraperitoneal perfusion with a 1% sodium bicarbonate solution on mice with the ascitic form of Ehrlich carcinoma in two series of experiments (27, 28). Ehrlich ascites carcinoma is a spontaneous murine mammary adenocarcinoma adapted to ascites form and carried in outbred mice by serial intraperitoneal injections (29). This model is well suitable for research on malignant ascites and cancer in general (30).

### 2.1 Materials and methods

#### 2.1.1 Animal studies

The studies were conducted on sexually mature female outbred laboratory mice (ICR/CD-1) obtained from the same nursery. All animals were clinically healthy and examined by a veterinarian. The animals were housed in similar vivarium conditions, with seven animals per cage, under a 12-hour day-night cycle at a temperature of 22°C, and received a standard diet. In both studies, three groups were randomly formed: control (n=14), pH+ (n=14), and pH- (n=14). At the start of the experiment, in the first study, animals in all groups were intraperitoneally injected with 0.6**·**10^5^ Ehrlich carcinoma cells suspended in 0.2 ml of PBS solution. In the second study, the number of transplanted cells was increased to accelerate the rate of tumor development and animals in all groups were intraperitoneally injected with 12**·**10^5^ Ehrlich carcinoma cells suspended in 0.2 ml of PBS solution. In both studies, at day 7 after tumor injection, mice were intraperitoneally perfused with different solutions. In the pH- group, they were perfused with 0.9% sodium chloride solution (pH~5.5), while in the pH+ group, they were perfused with 1% sodium bicarbonate solution/0.675% sodium chloride solution (4% sodium bicarbonate solution was 4-fold diluted with 0.9% sodium chloride solution; pH~8.2). The mice in control group were intact.

The perfusion procedure was performed on animals under anesthesia. We selected the concentration and duration of incubation with sodium bicarbonate solution in healthy animals to avoid alkalosis toxicity. The perfusion procedure involved the following steps: 1. Tumor ascites evacuation; 2. Intraperitoneal administration of 10 ml perfusate solution with a 10-minute incubation period; 3. Evacuation of the perfusate solution; 4. Repeating of steps 2 and 3; Intraperitoneal washing with 10 ml of a 0.9% sodium chloride solution. In the second study, one random animal from each group was euthanized to conduct experiments with ascites fluid on day 7, 14, and 18 from the start of the experiment. The pH measurements of the ascites fluid were conducted using a pHep+ pH meter (Hanna, Germany) and pH-indicator strips, specifically the pH 5.0 - 10.0 test strips (Merck Millipore, Germany). At day 32, the remaining 3 animals in group pH+ were euthanized, and a macroscopic examination of the peritoneal cavity was performed.

Statistical data processing was carried out using R language software in the RStudio environment. Kaplan-Meier method and log-rank test were applied to determine animal survival. The critical level of significance when testing statistical hypotheses was accepted as p ≤ 0.05.

#### 2.1.2 Cell cycle, viability and apoptosis analysis

Ascites fluid cell concentration were measured using the Countess II Automated Cell Counter (Thermo Fisher Scientific, USA). To determine cell viability and apoptosis a FITC-Annexin V apoptosis detection kit (BD Pharmingen, USA) and Propidium Iodide (PI, Sigma-Aldrich, USA) were used. Briefly, the ascites fluid cell suspension was vortexed for 15 s, centrifuged for 5 min at 400 g, the supernatant was aspirated and cells washed once with cold phosphate-buffered saline (PBS). After these cells were resuspended in 100 µl 1X Annexin V binding buffer and 5 µl of FITC Annexin-V and 10 µl of PI were added to the cell suspension. The cells were incubated for 20 min at room temperature in the dark, and then 400 μl of binding buffer was added to each tube. Stained cells were analyzed using an EPICS XL flow cytometer (Beckman Coulter, USA) with excitation at 488 nm and measuring green fluorescence emission for FITC Annexin V and red fluorescence emission for Propidium Iodide.

For cell cycle analysis, cell samples were washed three times with phosphate-buffered saline (PBS) and then added with 0.2 mg/ml saponin (Sigma, USA), 0.25 mg/ml RNase (Sigma, USA) and 0.05 mg/ml PI. After 30 min incubation at room temperature, cell samples were analyzed on an EPICS XL flow cytometer (Beckmann Coulter, USA). Data processing was performed using ModFit LT software (Verity Software House, Topsham).

Results were statistically processed using MaxStat 3.06 software (MaxStat Software, Germany). All data from three independent experiments were measured with standard error of the mean (mean ± SE) compared using Student’s t-test or U-Wilcoxon-Mann-Whitney nonparametric test. Differences between groups were considered significant at p ≤ 0.005.

### 2.2 Results

In the first study, we observed a significant extension in the overall survival of the mice treated with sodium bicarbonate solution compared to those treated with a 0.9% sodium chloride solution. Specifically, the median survival period of the sodium bicarbonate group was 24 days, while that of the sodium chloride or control group was 17 days (p<0.05) (data not shown) (27). To confirm this result, we repeated the experiment (28), increasing the initial number of transplanted Ehrlich carcinoma cells by a factor of two compared with the first study (12·10^5^ vs. 6·10^5^).

**Figure 1:**
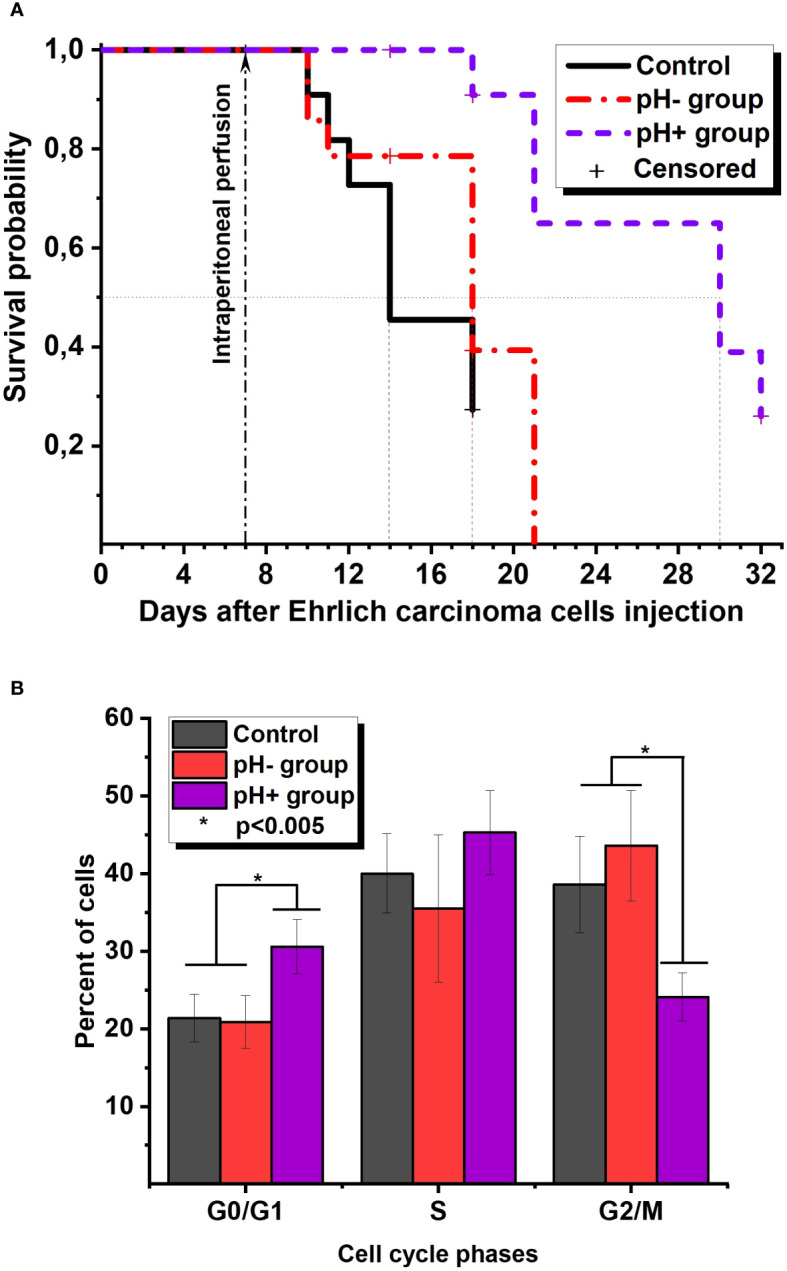
**(A)** The survival Kaplan-Meier curve for Ehrlich ascites carcinoma mice. **(B)** Ehrlich ascites cells cycle phases distribution on the 11th day after perfusion.

**Figure 1A** represents the survival Kaplan-Meier curve for Ehrlich ascites carcinoma mice. The survival analysis showed that in the pH+ group, mice had a median survival of 30 days after tumor cell injection, which was significantly different from the median survival of 18 days in the pH- group and 14 days in the control group (p<0.05). Censored points mean that one random animal from each group was euthanized to conduct experiments with ascites fluid on this day. The measured ascitic fluid pH values were 6.9 ± 0.1 in all groups. The exception is day 32, when the remaining 3 animals in group pH+ were euthanized, and a macroscopic examination of the peritoneal cavity was performed, which did not reveal signs of ascites. We did not observe any differences in ascites fluid cell concentration for groups in the experiment (data not shown). But it should be noted that we cannot differentiate cancer cells from other cells such as NK cells or T cells within the total ascites cell population. Flow cytometry measurements revealed that the percentage of apoptotic and dead cells was also equal in all groups. However, on the 11th day after the perfusion procedure, there was a difference in the percentage of cells in the G2/M, G0/G1, and S phases: 24.1 ± 3.1%, 30.6 ± 3.5%, 45.3 ± 5.4% in the pH+ group vs. 43.6 ± 7.1%, 20.9 ± 3.4%, 35.5 ± 9.5% in the pH- group, respectively (**Figure 1B**). In other words, in pH+ group we observed a significant increase in the number of cells in G0/G1 phase and a decrease in the number of cells in G2/M phase. So, we may suggest that a single intraperitoneal perfusion with a sodium bicarbonate solution has some antiproliferative effect. Although the mechanisms underlying such antitumor activity of sodium bicarbonate in the case of Ehrlich ascites remain unclear, we assume that the results of preclinical studies can be translated to the clinic.

## 3 Malignant ascites therapy with sodium bicarbonate: a case study of ovarian cancer ascites

According to preliminary results from some studies, ovarian cancer ascitic fluid pH is generally alkaline (pH>7.3) (31, 32). Despite this, intraperitoneal perfusion with an alkalizing solution could still have an effect. It would further extremely increase the extracellular pH for floating complexes of adenocarcinoma cells and would also influence the solid component of peritoneal carcinomatosis, which is the source of these cells.

### 3.1 Case description

**Figure 2:**
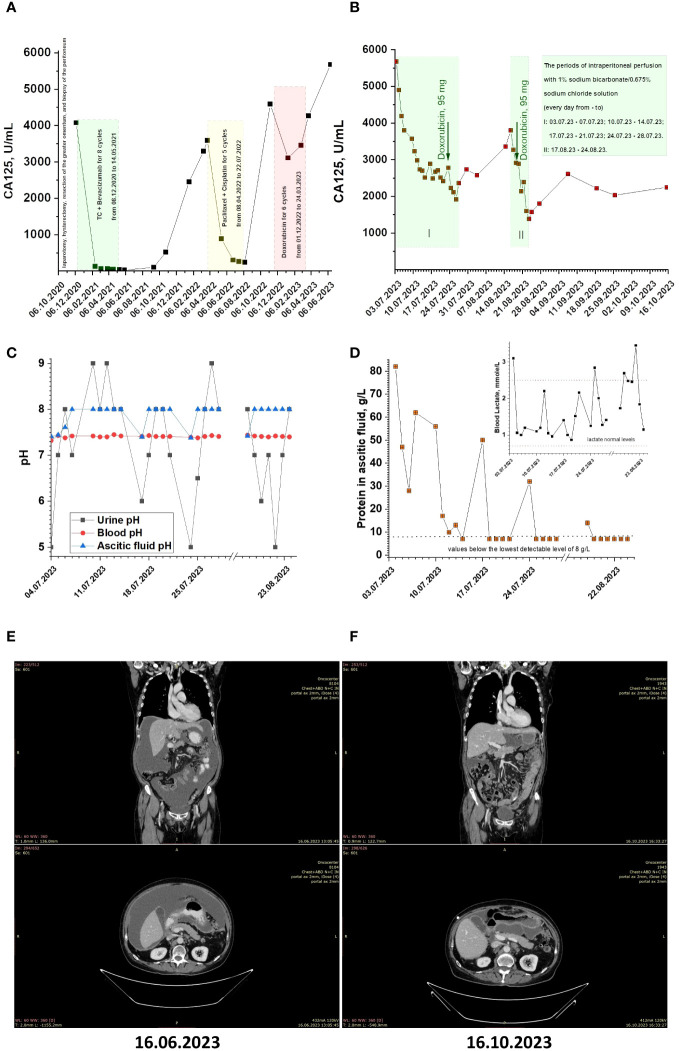
**(A, B)** CA125 monitoring for patient under conventional and alkalization treatments. **(C)** The pH values of patient’s ascitic fluid, blood and urine during alkalization treatment. **(D)** The protein concentration in patient’s ascitic fluid during alkalization treatment (Inset. Patient’s blood lactate levels during alkalization treatment). **(E, F)** Patients CT scans before and after alkalization treatment.

The patient in the case study was a 71-year-old female. In 2014, the patient underwent bilateral adnexectomy due to serous cystadenoma of the ovaries, vaginal plastic surgery, and ureteroplasty. In September 2020, at our Oncocentre, the patient underwent puncture of the posterior vaginal vault, which revealed glandular cancer cells. In October 2020, the patient underwent laparotomy, hysterectomy, resection of the greater omentum, and biopsy of the peritoneum. The histological result was high-grade serous ovarian carcinoma. Subsequently, the patient underwent eight cycles of polychemotherapy (ТС + Bevacizumab) from 08.12.2020 to 14.05.2021, and the treatment was well-tolerated. In March 2022, a CT scan revealed peritoneal carcinomatosis, ascites, and lymphadenopathy. The patient underwent laparocentesis and started the second-line chemotherapy regimen of Paclitaxel + Cisplatin for 5 cycles from 08.04.2022 to 22.07.2022. In November 2022, a CT scan revealed an increase in peritoneal carcinomatosis and ascites. The patient commenced the third-line chemotherapy regimen of doxorubicin for 6 cycles from 01.12.2022 to 24.03.2023. As of March 2023, the patient has undergone six cycles of third-line chemotherapy and has shown disease stabilization. The patient’s CA-125 levels have been monitored throughout treatment (**Figure 2A**). In June 2023, the patient presented to our Oncocentre with progressive ascites. CA125 level was more than 5500 U/ml. CT scan revealed progression of peritoneal carcinomatosis, ascites, and lymphadenopathy (**Figure 2E**). With treatment options largely exhausted, the patient agreed to experimental alkalization therapy using sodium bicarbonate.

On June 30, 2023, the patient underwent implantation of a peritoneal port-catheter system under ultrasound and X-ray control. The patient received a series of intraperitoneal perfusions with a solution containing 1% sodium bicarbonate and 0.675% sodium chloride (4% sodium bicarbonate solution was 4-fold diluted with 0.9% sodium chloride solution; pH~8.2). The perfusion procedure involved the following steps: 1. Tumor ascites evacuation; 2. Intraperitoneal administration of 1400 ml perfusate solution; 3. Evacuation of the perfusate solution; 4. Repeating of steps 2 and 3; Intraperitoneal washing with 1400 ml of a 0.9% sodium chloride solution. The perfusions were administered daily during the following periods: 03.07.23 - 07.07.23; 10.07.23 - 14.07.23; 17.07.23 - 21.07.23; 24.07.23 - 28.07.23.

The patient’s CA-125 levels have been monitored almost daily throughout treatment (see **Figure 2B**). During the course of therapy, it is noteworthy that CA125 levels have significantly dropped from the beginning and have stabilized at around 2500 U/mL. This was a positive indication of the effectiveness of the treatment and suggests that the patient is responding well to therapy. In an attempt to restore the effect of doxorubicin through the ‘ion trapping’ phenomenon (11, 20, 21), the patient received a dose of 60 mg/m^2^ alongside alkalization during the last perfusion period. After this, the CA125 level dropped to 1971 U/mL, and the patient was sent home to rest for three weeks. Upon return, the CA125 level increased to 3800 U/mL. The patient received a second series of daily peritoneal perfusions from 17.08.23 to 24.08.23, and a second reintroduction of doxorubicin 19.08.23. After this, the CA125 level dropped to around 1600 U/mL. After the course of therapy, the patient was sent home with periodic monitoring of CA125 levels and condition.

Note that, in addition to the higher alkalization of ascitic fluid (pH value of ascitic fluid before the start of therapy: 7.41), we observed systemic alkalization, weakly manifested in terms of changes in blood pH, but clearly manifested in changes in urine pH (urine pH value before therapy: 5) (**Figure 2C**). Also, during therapy, a decrease in protein concentration in ascitic fluid below the detection limit (less than 8 g/mL) was observed. Additionally, during therapy, blood lactate concentrations returned to normal values (blood lactate concentration before therapy was 3.1 mmol/l) (**Figure 2D**, inset). At the same time, median value of blood glucose was 6.295 mmol/L.

Over approximately 2 months of observation, the CA125 level remained at about 2200 U/mL (**Figure 2B**). A control CT scan showed stabilization of peritoneal carcinomatosis and lymphadenopathy process and disappearance of ascites (**Figure 2F**).

## 4 Discussion

Our obtained preclinical and clinical results are encouraging, although they require an explanation of the mechanisms of the antitumor effect of sodium bicarbonate. First, it is worth noting that using other alkalizing agents, such as sodium citrate or dichloroacetate, can have an antitumor effect. For instance, sodium citrate, an anti-glycolytic agent, was found to reduce the expression level of Mcl-1 in ovarian carcinoma cells. Furthermore, the continuous exposure of these cells to sodium citrate demonstrated a dose-dependent cytotoxic effect (33). The antitumor action of dichloroacetate is associated with its effect on increasing the activity of pyruvate dehydrogenase. Dichloroacetate has been found to alter glucose metabolism, pH homeostasis, and cell survival regulation (34). Based on the analysis of other existing studies, the following careful assumptions can be made.

We can assume that in our clinical case, the ‘ion trapping’ effect worked, and the inhibitory concentration for doxorubicin decreased. That is, alkalization helped to at least partially overcome the already formed insensitivity to this chemotherapy weak base drug. Also it was shown recently that the alkalization of the tumor microenvironment with sodium bicarbonate leads to even greater intracellular alkalization of tumor cells and their death (35). In other words, the bicarbonate ion, which influences intracellular pH, can trigger a cascade of molecular events leading to the death of tumor cells. Specifically, alkalinization of intracellular pH with sodium bicarbonate reduces the pH gradient, membrane potential, and proton motive force at the inner mitochondrial membrane. The disruption of OXPHOS due to the collapse of proton motive force leads to a significant increase in adenosine monophosphate (AMP), which activates AMP-activated protein kinase-mediated autophagy. However, the autophagy process is ultimately blocked by increased intracellular pH, decreased OXPHOS, and inhibition of the lysosomal proton pump under alkaline conditions. Sodium bicarbonate also induces sustained mitochondrial permeability and damages mitochondria (35).

This conception of sodium bicarbonate cytotoxic action is in a good agreement with novel type of cell death – alkaliptosis. Alkaliptosis (a pH-dependent form of regulated necrosis) is a recently discovered form of regulated cell death driven by intracellular alkalization (36). It is a pH-dependent cell death mechanism that has been found to be effective in tumor therapy, particularly in pancreatic ductal adenocarcinoma (PDAC) cells (37). Alkaliptosis is characterized by intracellular alkalization and does not require the activation of traditional cell death effectors such as caspases, MLKL, and toxic lipids. The underlying molecular mechanisms and regulatory networks of alkaliptosis are still largely unknown. However, recent studies have identified several key regulators and pathways involved in alkaliptosis. These include the acetate-activating enzyme ACSS2 (38), ATP6V0D1 (39), HMGB1 (40), and NF-κB activation (41). Understanding the mechanisms of alkaliptosis may provide new insights for developing targeted therapies for cancer treatment. However, it is important to note that intracellular alkalization with sodium bicarbonate can lead to the same cell death as chemically induced alkaliptosis. Therefore, the preclinical and clinical effects we observed may be a result of the direct cytotoxic antitumor effect of sodium bicarbonate.

Next, lactate, a byproduct of glycolysis, has been recently found to regulate the cell cycle by remodeling the anaphase promoting complex (APC/C), which stimulates the timed degradation of cell cycle proteins and efficient mitotic exit in proliferative human cells. Accumulated lactate has been shown to directly regulate protein function to control the cell cycle and proliferation, communicating the consequences of a nutrient-replete growth phase to stimulate cell division and proliferation (42). Sodium bicarbonate, or alkalization, reduces lactate-induced acidosis and leads to lactosis (predominantly, the salts of lactic acid with ions such as sodium, potassium, etc. exist, rather than lactic acid itself). This means that the concentration of protons decreases, which in turn reduces lactate transport through monocarboxylate transporters and disrupts the joint action of lactate and proton (43). The shift in the cycle toward the G0/G1 phase, obtained in preclinical studies for mice with Ehrlich ascites carcinoma after intraperitoneal perfusion with a sodium bicarbonate solution, can be explained by these facts.

Moreover, there is evidence that by disrupting the joint action of lactate and proton through bicarbonate infusion into tumors, the glycolytic rate of cancer cells relying on glycolysis was maximized. This led to the depletion of intratumoral glucose and exposed cancer cells to glucose deprivation, ultimately resulting in their death (12, 43). In our clinical case, we observed a rapid normalization in blood lactate after a sodium bicarbonate perfusion procedure. Also, sodium bicarbonate can re-energize metabolically impaired T cells and reverse the inhibitory effects of lactic acid on T cells (24). The antitumor effect of sodium bicarbonate may be linked to improved immune response. And of course, along with the above, we should not exclude the possibility that washing of the peritoneal cavity contributed to a decrease in the ascites concentration of “pathogenic” proteins (44), such as CA125, which promotes ovarian tumor growth and metastasis (45) and protects ovarian tumor cells from recognition by NK cells (46). It is worth noting that proteins can change their conformation, functionality, and even denature with extreme changes in pH. The isoelectric point of the CA125 protein is almost near 7.3 (pI = 6.2–7.3) (47), so a pH of 8 can also lead to its degradation, as can be the case for other “pathogenic” proteins. Our study demonstrated a total decrease in ascitic fluid protein levels and a significant decrease in CA125 blood levels. Therefore, possible protein degradation may be a part of the alkalization therapeutic effect.

These assumptions may explain the preclinical and clinical results obtained using sodium bicarbonate perfusion in the treatment of malignant ascites. Undoubtedly, these results represent a small yet significant contribution to the evolving field of tumor alkalization as a cancer therapy, and they unequivocally affirm the potential of this concept.

## Data availability statement

The original contributions presented in the study are included in the article/supplementary material. Further inquiries can be directed to the corresponding author.

## Ethics statement

The studies involving humans were approved by local ethics committee, Napalkov Saint Petersburg Clinical Research and Practical Center of Specialized Types of Medical Care (Oncological). The studies were conducted in accordance with the local legislation and institutional requirements. The participants provided their written informed consent to participate in this study. The animal study was approved by local ethics committee, Napalkov Saint Petersburg Clinical Research and Practical Center of Specialized Types of Medical Care (Oncological). The study was conducted in accordance with the local legislation and institutional requirements. Written informed consent was obtained from the individual(s) for the publication of any potentially identifiable images or data included in this article.

## Author contributions

AlB: Conceptualization, Data curation, Formal analysis, Investigation, Methodology, Project administration, Supervision, Visualization, Writing – original draft, Writing – review & editing. NVe: Data curation, Investigation, Methodology, Writing – review & editing. AnB: Data curation, Investigation, Visualization, Writing – original draft, Writing – review & editing. VB: Investigation, Writing – review & editing. VS: Data curation, Investigation, Writing – review & editing. VE: Data curation, Methodology, Project administration, Writing – review & editing. NVo: Data curation, Methodology, Project administration, Writing – review & editing. VM: Conceptualization, Data curation, Formal Analysis, Methodology, Project administration, Supervision, Writing – review & editing.
